# Benzene exposure and health risk assessment among workers in informal footwear workshops: A cross-sectional study in Medan, Indonesia

**DOI:** 10.1016/j.toxrep.2025.102140

**Published:** 2025-10-11

**Authors:** Taufik Ashar, Devi Nuraini Santi, Evi Naria

**Affiliations:** Department of Environmental Health, Faculty of Public Health, Universitas Sumatera Utara, Medan 20155, Indonesia

**Keywords:** Benzene, Occupational exposure, Biomarker, Health risk, Tt-MA, MDA, Footwear workshop

## Abstract

Benzene is widely used in small-scale industries, particularly in informal footwear manufacturing, due to its adhesive properties. However, its volatility and carcinogenicity pose serious health risks to workers in poorly regulated environments. This study hypothesized that informal footwear workers in Medan are exposed to benzene concentrations exceeding international safety standards, resulting in measurable biological effects and increased health risks. Environmental monitoring was conducted in seven workshops, revealing ambient benzene levels ranging from 0.101 to 0.5147 mg/m³ . Five locations exceeded the NIOSH Recommended Exposure Limit (0.32 mg/m³), and all surpassed the WHO air quality guideline (0.005 mg/m³). Biomarker analysis showed elevated trans,trans-muconic acid (tt-MA) and malondialdehyde (MDA) levels, particularly in workshops with poor ventilation and high worker density. A moderate correlation between tt-MA and MDA (r = 0.462, p = 0.003) confirmed the link between benzene exposure and oxidative stress. Clinical symptoms such as headache and appetite loss were significantly associated with MDA and tt-MA levels, respectively, suggesting early biological effects. Carcinogenic risk assessment indicated that four locations exceeded acceptable lifetime cancer risk thresholds, with Location VI presenting a 65-fold exceedance. These findings underscore the urgent need for low-cost interventions, such as improved ventilation and safer chemical substitutes, to reduce exposure in informal industrial settings. Longitudinal studies are recommended to clarify causal pathways and inform occupational health policy.

## 1 Introduction

Benzene, a volatile organic compound (VOC), is a well-established occupational hazard due to its hematotoxic and carcinogenic properties, classified as a Group 1 carcinogen by IARC [1]. While exposure is regulated in formal industries, a significant knowledge gap exists in the informal sector, where regulatory oversight and occupational safeguards are often lacking [2]. This deficit is particularly pronounced in developing nations across Southeast Asia, including Indonesia, where informal economies constitute a substantial portion of the workforce [3], [4].

Among various informal industries, small-scale footwear manufacturing workshops represent a uniquely high-risk environment. Unlike sectors with intermittent exposure such as automotive repair [5], [6] or printing [7], footwear production involves the intensive and continuous use of solvent-based adhesives in poorly ventilated, confined spaces [8], [9]. This specific work practice is expected to result in higher and more sustained benzene concentrations, yet empirical data to confirm this risk, particularly from Southeast Asia, remain sparse. The absence of robust, localized evidence hinders the development of effective public health interventions and tailored occupational safety policies for this vulnerable population.

To address this gap, emerging research advocates for an integrated approach that combines environmental monitoring with biological monitoring to achieve a more holistic assessment of exposure and its subclinical effects [10], [11]. Urinary *trans,trans*-muconic acid (tt-MA), a metabolite of benzene, serves as a well-validated biomarker of internal dose [12], [13], [14]. Concurrently, malondialdehyde (MDA), a byproduct of lipid peroxidation, provides a sensitive measure of systemic oxidative stress, a key mechanism in benzene-induced toxicity [15], [16], [17]. The concurrent measurement of these biomarkers enables a more nuanced evaluation that surpasses the limitations of relying on ambient air monitoring alone [18].

Therefore, this study was designed to test the central hypothesis that workers in informal footwear workshops in Medan, Indonesia, are exposed to hazardous benzene levels that result in unacceptable health risks, and that this exposure is associated with a cascade of measurable biological effects**.** Specifically, we aimed to: (1) quantify ambient benzene concentrations and assess the corresponding non-carcinogenic and carcinogenic health risks; (2) measure urinary tt-MA and MDA levels as biomarkers of exposure and oxidative stress; and (3) investigate the correlation between exposure levels, biomarker responses, and self-reported health symptoms. By integrating these multi-level assessments, this research provides novel insights into the health impacts of benzene in a critically under-researched sector and offers actionable evidence to inform targeted workplace interventions.

## 2 Methods

### 2.1 Study design and hypotesis

This cross-sectional study was conducted between April and October 2017 in seven informal footwear workshops in Medan, Indonesia. The central hypothesis was that workers are exposed to benzene concentrations exceeding international safety thresholds, resulting in measurable biological effects specifically elevated urinary biomarkers (tt-MA and MDA) and increased health risks. These endpoints were selected to capture both internal dose and early biological response, enabling a comprehensive assessment of exposure impact.

### 2.2 Study population

A total of 40 workers were purposively recruited from an estimated workforce of 120. This sample size (33 %) was considered adequate for exploratory analysis in informal settings, where participation is often constrained by regulatory concerns and logistical limitations [8], [9]. Inclusion criteria required ≥ 1 year of continuous employment and daily exposure to benzene-containing adhesives for ≥ 8 h per shift. Exclusion criteria included chronic illness or antioxidant intake within 24 h prior to sampling, to minimize confounding in oxidative stress biomarkers.

### 2.3 Environmental monitoring

Ambient benzene concentrations were measured using personal air sampling (NIOSH Method 1501) [19], with SKC pumps (0.2 L/min, 8-hour duration) and activated charcoal tubes. Samples were analyzed via GC-FID after desorption with carbon disulfide. The method’s limit of detection (LOD) was 0.01 mg/m³ and limit of quantification (LOQ) was 0.03 mg/m³ . Room dimensions, ventilation area, and worker density were recorded to assess exposure determinants.

### 2.4 Biological Monitoring

Post-shift urine samples (≥10 mL) were collected and stored at −20°C. Urinary creatinine was measured enzymatically using the Jaffé method [20] to normalize biomarker levels. Benzene exposure was assessed via trans,trans-muconic acid (tt-MA) using HPLC-UV [21], with LOD 50 µg/L and LOQ 150 µg/L. Oxidative stress was evaluated via malondialdehyde (MDA) using the TBARS assay [15], with LOD 0.1 µmol/L and LOQ 0.3 µmol/L. Biomarkers were expressed as µg/g creatinine (tt-MA) and µmol/mol creatinine (MDA).

### 2.5 Anthropometric and occupational data

Anthropometric data, including body weight, were measured using calibrated digital scales. Structured interviews were conducted to collect data on demographics, occupational history, work practices, and self-reported health symptoms. Smoking status was recorded for all participants as part of the demographic and lifestyle data collection.

### 2.6 Risk assessment method

Health risk assessment was conducted following USEPA guidelines to quantify both non-carcinogenic and carcinogenic risks associated with measured benzene exposure.

#### 2.6.1 Non-carcinogenic risk assessment

Non-carcinogenic risk was estimated using the Hazard Quotient (HQ), calculated as the ratio of chronic daily intake (CDI) to the reference concentration (RfC = 0.03 mg/m³) [22]. The inhalation exposure dose of benzene was calculated using the standard formula recommended by the USEPA for non-carcinogenic risk assessment [23]:

$${\mathrm{CDI}}_{\mathrm{inhalation}}=\frac{(C\;\times \mathrm{IR}\times \mathrm{ET}\times \mathrm{EF}\times \mathrm{ED})\;}{\left(\mathrm{BW}\times \mathrm{AT}\right)}/$$

where CDI_inhalation is the chronic daily intake of contaminate via inhalation (mg·kg⁻¹·day⁻¹), C is the concentration of benzene in air (mg·m⁻³), IR is the inhalation rate (m³·h⁻¹), estimated average lifetime inhalation rates for males and females are 14 m³ ·day⁻¹ and 10 m³ ·day⁻¹ [18], ET is the exposure time (h·day⁻¹) (the frequency of exposure h per day, 8 h based on actual working hours), EF is the exposure frequency (days per year), the frequency of exposure (300 working days in a year = 6 days/week × 50 weeks/year), and ED is the exposure duration (years), which is defined as the time that an individual or population is exposed to contaminants and probably becomes ill.

The exposure duration assumptions are based on USEPA guidelines for occupational risk assessment: for non-carcinogenic effects, a 25-year working period is used as the standard occupational exposure duration, representing a typical career span. This conservative approach is appropriate even though many current workers have shorter employment histories, as it estimates potential risks for workers who may continue in this occupation long-term. This standardized approach does not reflect individual current work tenure but rather provides a consistent framework for regulatory risk assessment and policy development. Additionally, BW is bodyweight (kg) and AT includes the number of days a person lives; for the risk assessment of non-cancer diseases, AT is average time in days per exposure period (25 years for general working period is equivalent to 9125 days) guided by the USEPA [23]. In this study, data regarding the duration of exposure, body weight, length of exposure period, and frequency of exposure were gathered using a questionnaire designed by the researcher.

Non-cancer risk is displayed by HQ. The HQ is the ratio between the actual exposure dose and the reference concentration considered safe. RfC_i for benzene is RFC = 0.03 mg/m³ [22]. HQ was calculated using the following formula:

$${\mathrm{HQ}}_{\mathrm{inhalation}}=\frac{\mathrm{CDI}\mathrm{inhalation}}{\mathrm{RFC}\mathrm{inhalation}}$$

An HQ value ≤ 1 indicates that exposure is within the safe limit and unlikely to cause systemic toxic effects in humans. Conversely, an HQ > 1 suggests that exposure exceeds the recommended threshold and requires further mitigation measures.

#### 2.6.2 Carcinogenic risk assessment

The following equation is used to calculate Lifetime Cancer Risk (LCR):

LCR = IUR × CDI_inhalation_

where IUR (Inhalation Unit Cancer Risk) = 2.2 × 10⁻⁶ to 7.8 × 10⁻⁶ per µg/m³ . If the risk value > IUR or 2.2 × 10⁻⁶ to 7.8 × 10⁻⁶ per µg/m³ , that means an unacceptable risk concerning cancer [21]. A 70-year lifetime (25,550 days) was assumed, with occupational exposure spanning 25 years, consistent with USEPA standards for genotoxic carcinogens. This conservative approach enables comparison with regulatory thresholds and reflects potential cumulative risk for workers in informal sectors.

### 2.7 Statistical analysis

Data were analyzed using SPSS v21. Normality was assessed via Shapiro-Wilk test. Descriptive statistics summarized demographic and exposure data. Chi-square tests examined symptom associations; *t*-tests or Mann-Whitney U tests compared biomarker levels. Spearman’s correlation assessed relationships between airborne benzene and urinary biomarkers. All tests were two-tailed with significance set at p < 0.05. Ethical approval and informed consent were obtained in accordance with the Declaration of Helsinki.

## 3 Result and discussion

### 3.1 Demographic characteristics of workers

Table 1 presents the demographic profile of the 40 occupational workers recruited from seven small-scale shoe workshops in Medan City.

**Table 1:** Demographic Characteristics of Workers in Seven Informal Footwear Workshops.

**Demographic Characteristics**	**n (%)**
Sex, n (%)	
Male	39 (97,5)
Female	1 (2,5)
Age, n (%)	
< 20 years	1 (2,5)
20 – 39 years	21 (52,5)
40 – 59 years	15 (37,5)
> 60 years	3 (7,5)
Education level, n (%)	
Elementary School	10 (25)
Junior High School	19 (47.5)
Senior High School School	11 (27.5)
Work Duration, n (%)	
< 5 years	29 (72,5)
≥ 5 years	11 (27,5)
Working Hours per day, n (%)	
≤ 8 h	22 (55)
> 8 h	18 (45)
Smoking Habbit, n (%)	
Yes	33 (82,5)
No	7 (17.5)

The demographic profile of 40 informal footwear workers in Medan reflects labor patterns typical of low- and middle-income countries (LMICs). Male dominance (97.5 %) aligns with gendered divisions in Indonesia’s footwear industry, where men perform physically demanding tasks and socio-cultural norms limit female participation [3], [4]. Most workers (90 %) were aged 20–59 years, representing the economically active population vulnerable to chronic health effects from prolonged benzene exposure, including hematologic and respiratory disorders [24], [25].

Educational attainment was low, with only 27.5 % completing senior high school. Limited formal education may impair health literacy and reduce adherence to safety practices, as shown in informal sectors lacking structured training [26], [27]. Passive safety communication is often ineffective, underscoring the need for tailored health education strategies [28].

Work tenure data revealed that 72.5 % had < 5 years of employment, suggesting job instability, while 27.5 % faced greater cumulative exposure risks due to longer service in poorly controlled environments. Chronic low-dose benzene exposure has been linked to hematotoxicity and increased cancer risk, even below regulatory thresholds [29], [30], [31]. Extended working hours were common (45 % >8 h/day), potentially intensifying exposure and contributing to fatigue, reduced vigilance, and immune suppression in poorly ventilated settings [32].

Smoking prevalence was high (82.5 %), presenting a confounding factor in tt-MA biomonitoring, as tobacco smoke is a known source [10], [11], [33]. Nonetheless, consistent tt-MA levels and ambient air data suggest occupational exposure remains the primary contributor. Cotinine-adjusted models are recommended to differentiate lifestyle and workplace exposures.

Overall, the convergence of low education, long shifts, high smoking rates, and lack of regulatory oversight places these workers at elevated risk for occupational disease. Targeted interventions and longitudinal surveillance are essential to reduce health burdens in informal industrial clusters such as Medan.

### 3.2 Workplace environmental characteristics and benzene exposure

Table 2 summarizes the environmental conditions and ambient benzene levels across seven footwear workshops in Medan. Informal footwear workshops in Medan operate in confined spaces with poor ventilation and rely on benzene-containing adhesives—a known carcinogen [1]. Ventilation-to-room area ratios were generally low (<10 % in five of seven locations), limiting air exchange and facilitating VOC accumulation [6]. An inverse relationship was observed between ventilation efficiency and benzene concentration, with Location I (22.86 %) recording the lowest level (0.101 mg/m³) and Location VI (4.68 %) the highest (0.5147 mg/m³) [34].

**Table 2:** Workplace Environmental Characteristics and Ambient Benzene Concentrations Across Study Locations.

**Location**	**I**	**II**	**III**	**IV**	**V**	**VI**	**VII**
Number of Workers	6	7	12	2	6	4	3
Room Area, m^2^	28	21.6	88	49	28	25	28
Ventilation Area, m^2^	6.4	1.98	4.4	6.04	2.31	1.62	1.62
Ventilation to Room Area Ratio, %	22.86	9.17	5	12.33	8.25	4.68	5.79
Mechanical Ventilation	-	-	-	+	-	-	-
Benzene Concentration. mg/m^3^	0.101	0.251	0.1460	0.1778	0.4267	0.5147	0.2867

Measured benzene concentrations ranged from 0.101 to 0.5147 mg/m³ . Two workshops (V and VI) exceeded the NIOSH REL of 0.32 mg/m³ [1], indicating localized overexposure. Compared to the WHO guideline (0.005 mg/m³ annual average) [8], all workshops recorded levels 20–100 times higher, highlighting widespread and severe exposure risks in unregulated micro-industrial settings.

Even Workshop IV, equipped with mechanical ventilation, showed elevated benzene (0.1778 mg/m³), suggesting design or maintenance deficiencies. These findings underscore the inadequacy of current ventilation practices and the need for engineering controls tailored to high-emission environments.

High worker density in small rooms (e.g., Location II: 7 workers in 21.6 m²) further exacerbates exposure [35], [36], [37]. The absence of PPE, health surveillance, and regulatory oversight compounds vulnerability, reinforcing the urgency of targeted interventions to prevent occupational disease.

### 3.3 Non-carcinogenic and carcinogenic risk assessment

Table 3 presents both non-carcinogenic (HQ) and carcinogenic (LCR) risk assessments based on measured ambient concentrations, providing critical insights into the occupational health crisis facing informal footwear workers.

**Table 3:** Non-Carcinogenic and Carcinogenic Risk Assessment Results Across Study Locations.

**Loca** **tion**	**HQ Non-Carcinogenic**				**LCR**	
	**> 1**	**≤ 1**	**Mean±SD**	**Median** **(Min – max)**	**> 7.8 × 10**^**—6**^**per µg/m**^**3**^ **(unacceptable cancer risk)**	**≤ 7.8 × 10**^**-−6**^**per µg/m**^**3**^ **(acceptable cancer risk)**
I.	0	6 (100)	0,16 ± 0,08	0,16 (0,06 – 0,25)	4 (66,7)	2 (33,3)
II.	0	7 (100)	0,28 ± 0,06	0,27 (0,23 – 0,4)	7 (100)	0
III.	0	12 (100)	0,21 ± 0,08	0,21 (0,06 – 0,32)	11 (91,7)	1 (8,3)
IV.	0	2 (100)	0,31 ± 0,07	0,31 (0,26 – 0,36)	2 (100)	0
V.	1 (16,7)	5 (83,3)	0,88 ± 0,22	0,82 (0,66 – 1,3)	6 (100)	0
VI.	2 (50)	2 (50)	1,12 ± 0,75	1,19 (0,17 – 1,92)	4 (100)	0
VII.	0	3 (100)	0,38 ± 0,32	0,46 (0,03 – 0,64)	2 (66,7)	1 (33,3)

Location VI presented the highest health risk, with 50 % of samples exceeding the hazard quotient (HQ > 1; max HQ: 1.92), indicating potential for acute adverse effects. Its benzene concentration (0.515 mg/m³) surpassed the USEPA reference concentration (0.03 mg/m³) by 17-fold, likely contributing to hematological toxicity.

Location V also showed episodic overexposure, with 16.7 % of samples exceeding the non-carcinogenic threshold (HQ range: 0.65–1.30), likely during intensive adhesive use without protective measures. Such intermittent high-dose exposures may pose greater toxicological risks than continuous low-level exposure [29], [30], [31]

In contrast, Location I had the lowest mean HQ (0.16 ± 0.08), supported by the highest natural ventilation ratio (22.86 %), demonstrating the protective effect of simple architectural improvements [34]. Even Location IV, despite having mechanical ventilation, recorded a mean HQ of 0.31, suggesting suboptimal system design or maintenance. These findings highlight the need for targeted ventilation interventions in informal settings [8], [9].

### 3.4 Carcinogenic risk assessment

**Fig. 1:**
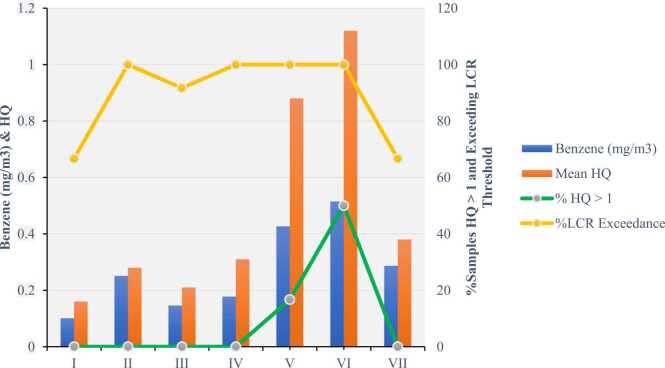
Combined visualization of benzene concentrations (mg/m³), mean Hazard Quotient (HQ), and the percentage of samples exceeding both the HQ threshold (>1) and the Lifetime Cancer Risk (LCR) threshold across seven informal footwear workshops in Medan.

**Fig. 2:**
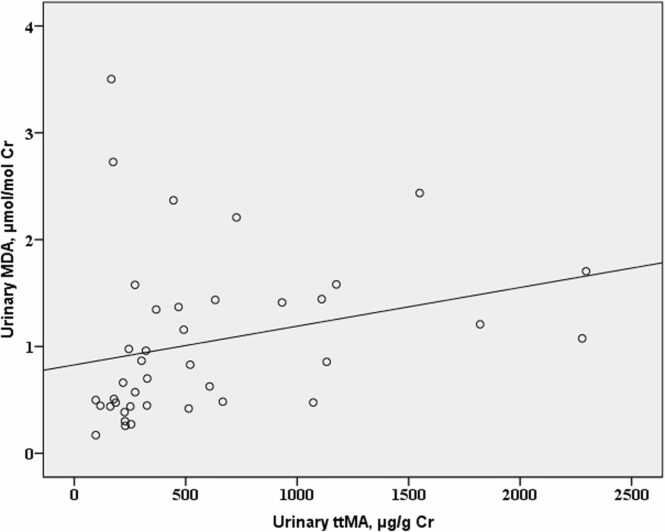
Scatterplot showing the correlation between urinary tt-MA and MDA levels (Spearman’s *r* = 0.462, *p* = 0.003).

Carcinogenic risk assessment indicates a serious public health concern in informal footwear workshops. Four locations (II, IV, V, VI) showed 100 % exceedance of acceptable cancer risk thresholds, with benzene concentrations ranging from 0.251 to 0.515 mg/m³ . These findings align with recent epidemiological evidence. Sassano et al. (2024) reported a significant association between occupational benzene exposure and colorectal cancer (pooled RR: 1.10, 95 % CI: 1.06–1.15), expanding the known carcinogenic profile beyond hematologic malignancies [38]. Wan et al. (2024) further identified links to lung cancer, suggesting that the overall cancer burden may be underestimated [39]. Fig. 1, Fig. 2

Location VI, with the highest concentration (0.515 mg/m³), presents an extreme cancer risk—exceeding acceptable lifetime thresholds by over 65-fold. This mirrors findings from prior assessments showing that workers in informal industries may face risks comparable to pre-regulation industrial settings [40].

Even moderate exposure levels warrant concern. Location III (0.146 mg/m³) showed 91.7 % exceedance, underscoring the relevance of the linear non-threshold (LNT) model, where cumulative risk persists even at low concentrations [41]. Young workers are particularly vulnerable, given the potential for prolonged exposure across their occupational lifespan.

### 3.5 Urinary ttMA and MDA levels

Table 4 presents the distribution of urinary ttMA and MDA among occupational workers in seven shoe workshops in Medan City, stratified by location. The majority of the workers were current smokers, with smoking prevalence ranging from 75 % to 100 % across locations, which is relevant given the potential influence of tobacco smoke on ttMA levels due to the presence of sorbic acid derivatives in mainstream smoke.

**Table 4:** Urinary ttMA and MDA Levels Among Workers by Study Locations.

**Loca** **tion**	**ttMA, µg/g Cr**				**MDA, µmol/mol Cr**	
	**> 500,** **n (%)**	**≤ 500,** **n (%)**	**Mean±SD**	**Min - Max**	**Mean±SD**	**Min - Max**
1	2 (33,3)	4 (66,7)	663,74 ± 822,91	96,65–2278,54	1,14 ± 0,94	0,17 – 2,37
2	1 (14,3)	6 (85,7)	347,03 ± 371,08	96,21–1175,84	1,21 ± 1,08	0,39–3,50
3	5 (41,7)	7 (58,3)	679,64 ± 638,48	178,31–2295,87	0,91 ± 0,69	0,27–2,44
4	2 (100)	0	796,04 ± 390,37	520–1072,07	0,65 ± 0,25	0,48–0,83
5	2 (33,3)	4 (66,7)	430,92 ± 379,2	117,23–1110,3	1,21 ± 0,89	0,26–2,73
6	2 (50)	2 (50)	888,25 ± 735,38	272,72–1820,56	1,09 ± 0,39	0,7–1,58
7	1 (33,3)	2 (66,7)	387,1 ± 211,16	186,40–607,35	0,82 ± 0,47	0,47–1,35

Note: Cr = creatinine; ttMA = trans,trans-muconic acid; MDA = malondialdehyde

Biomarker analysis confirmed substantial benzene exposure among informal footwear workers, with marked inter-location variability. Location VI recorded the highest mean tt-MA level (888.25 µg/g creatinine), followed by Location IV (796.04 µg/g creatinine), where all workers exceeded the recommended biological exposure limit of 500 µg/g creatinine [42]. These results align with prior studies indicating that benzene exposure in informal sectors can be 5–10 times higher than in regulated workplaces [43], driven by inconsistent work practices, poor ventilation, and individual metabolic differences [44].

The high variability in ttMA levels across workers in the same location particularly in Location 6 may reflect differences in proximity to benzene sources, duration of exposure, and individual metabolic rates. Furthermore, the presence of dietary sorbates, which can elevate ttMA levels, suggests that caution is needed in interpreting ttMA as a sole marker of occupational exposure [10], [11], [33].

Interestingly, oxidative stress markers (MDA) did not parallel tt-MA levels. Location II exhibited the highest MDA concentration (1.21 µmol/mol creatinine) despite lower benzene exposure, while Location IV showed the lowest MDA level (0.65 µmol/mol creatinine) despite high tt-MA values. This discrepancy suggests that oxidative damage is modulated by factors beyond benzene alone, including antioxidant capacity, genetic polymorphisms, and co-exposure to other chemicals [45], [46]. Lifestyle and physiological variables such as diet, age, and health status may further influence individual susceptibility to oxidative stress [47].

### 3.6 Smoking as a confounding factor

The high smoking prevalence (75–100 % across locations) presents an important consideration for interpreting tt-MA results. Tobacco smoke contains compounds that can be metabolized to tt-MA, potentially inflating exposure estimates [33]. However, the consistently elevated tt-MA levels in certain locations, particularly those with poor ventilation, suggest that occupational exposure remains the primary contributor to benzene burden in these workers.

Recent studies have developed methods to distinguish occupational from smoking-related benzene exposure using multiple biomarkers [48]. The correlation between ambient benzene levels and tt-MA in our study (r = 0.462, p = 0.003) supports the validity of tt-MA as an occupational exposure indicator, even in populations with high smoking rates. Table 5

**Table 5:** Correlation Between Urinary ttMA and Urinary MDA Among Workers.

	**Urinary MDA**	
	p*	**r**
**Urinary ttMA**	0003	0462

* Spearman Correlation

A moderate positive correlation between tt-MA and MDA levels (r = 0.462, p = 0.003) indicates that benzene exposure among informal footwear workers is biologically impactful, with elevated exposure linked to increased oxidative stress. This finding supports the biological plausibility of health risks and aligns with meta-analyses reporting similar correlation ranges (r = 0.3–0.6) between exposure and effect biomarkers [49]. Despite the limited sample size, the statistical significance suggests a robust and non-random association.

The mechanistic basis for this relationship is well-established: benzene metabolism in the liver produces reactive intermediates such as benzoquinones, which generate reactive oxygen species and induce lipid peroxidation, reflected by elevated MDA levels [50]. These insights are critical for guiding targeted occupational health interventions in high-exposure settings.

### 3.7 Health symptoms and biomarker associations

Table 6 presents the combined analysis of self-reported health symptoms and their associations with urinary biomarkers of exposure (tt-MA) and oxidative stress (MDA).

**Table 6:** Self-Reported Health Symptoms and Associated Biomarker Levels Among Workers.

**Health** **Symtoms**	**Cate-** **gory**	**n**	**Urinary ttMA,** **µg/g creatinine**	p*	**Urinary MDA, µmol/mol creatinine**	p*
Headache	Yes	19	467.93 (96.21–2295.87)	0.424	1.16 (0.26–3.5)	0.030
	No	21	301.76 (96.65–1820.56)		0.51 (0.17–2.37)	
Fatigue	Yes	8	763.96 ± 736.71	0.457	1.08 ± 0.58	0.521
	No	32	323.82 (96.21–2278.54)		0.68 (0.27–3.5)	
Loss of appetite	Yes	6	1380.64 ± 741.8	0.002	1.4 ± 0.24	0.025
	No	34	287.41 (96.21–1820.56)		0.64 (0.17–3.5)	
Skin irritation	Yes	11	273.6 (96.21–2295.87)	0.422	1 ± 0.58	0.596
	No	29	367.54 (96.65–2278.54)		0.83 (0.17–3.5)	
Eye irritation	Yes	5	821.53 ± 407.88	0.158	1.42 ± 0.64	0.130
	No	35	321.44 (96.21–2295.87)		0.7 (0.17–3.5)	
Sore throat	Yes	4	1058.96 ± 909.9	0.105	1.25 ± 0.51	0.191
	No	36	323.82 (96.21–2278.54)		0.77 (0.17–3.5)	
Shortness of breath	Yes	6	240.46 (166.28–2278.54)	0.405	1.53 ± 1.3	0.532
	No	34	347.42 (96.21–2295.87)		0.77 (0.17–2.44)	
Tremor	Yes	3	1125.68 ± 1069.34	0.457	2 ± 1.32	0.085
	No	37	326.2 (96.21–2295.87)		0.7 (0.17–2.73)	

Data are presented as mean ± SD and median (min–max)

*Statistical Analysis by Mann Whitney U Test

Headache emerged as the most common complaint, affecting nearly half of the workers (47.5 %), followed by skin irritation (27.5 %) and fatigue (20.0 %). This symptom pattern is consistent with established clinical presentations of chronic benzene exposure, where neurological and dermatological effects predominate [51], [52], [53]. The high prevalence of headache aligns with recent occupational health studies showing that central nervous system symptoms are early indicators of volatile organic compound exposure in poorly ventilated workplaces [54], [55].

The relatively high frequency of skin and eye irritation (27.5 % and 12.5 %, respectively) reflects direct contact with solvent vapors, which is common in informal industries where personal protective equipment use is limited [56]. These local irritant effects serve as immediate indicators of inadequate exposure control measures and highlight the need for improved workplace ventilation and protective practices.

### 3.8 Biomarker-symptom associations: key findings

Biomarker analysis revealed two key associations highlighting the biological impact of benzene exposure. Workers reporting headaches had significantly higher MDA levels (1.16 vs. 0.51 µmol/mol creatinine, p = 0.030), despite comparable tt-MA levels (p = 0.424), suggesting that oxidative stress rather than exposure intensity, may underlie neurological symptoms. This is supported by evidence that benzene-induced oxidative stress can impair neural function even at low exposure levels [51], with MDA emerging as a more sensitive marker than tt-MA for early neurotoxic effects.

Our findings revealed a significant association between chronic benzene exposure and loss of appetite, supported by elevated levels of both tt-MA and MDA among affected individuals. This suggests that benzene may disrupt neurochemical pathways involved in appetite regulation, potentially through oxidative stress mechanisms. Similar clinical patterns have been documented in case reports of benzene-induced toxic encephalopathy, where gastrointestinal symptoms such as appetite suppression preceded cognitive decline. These results reinforce the plausibility of appetite loss as an early, biologically meaningful symptom of chronic benzene toxicity [57]. However, it is important to note that the association between loss of appetite and elevated biomarker levels is based on a small subgroup (n = 6), and these findings should be interpreted with caution. While the consistency between biomarker elevations and clinical observations provides biological plausibility, larger studies are needed to validate this association and establish its clinical significance in occupational health surveillance.

### 3.9 Clinical implications and early detection

The observed biomarker-symptom associations offer valuable insights for occupational health surveillance in informal sectors. The specific correlation between headache and elevated MDA levels suggests that oxidative stress biomarkers may serve as early indicators of neurotoxicity, even under moderate exposure conditions. This is particularly relevant for settings lacking advanced monitoring, where simple biomarker screening could enhance early detection.

The moderate association between loss of appetite and both tt-MA and MDA levels supports its use as a clinical marker of significant benzene exposure. Unlike general symptoms such as fatigue, appetite suppression reflects a dose-response relationship that may guide intervention priorities in workplace health programs [58].

### 3.10 Limitations and future directions

This study offers valuable insights into benzene exposure in informal footwear workshops; however, several limitations must be acknowledged. The relatively small sample size and limited geographic coverage restricted to seven workshop locations in Medan, may constrain the generalizability of the findings. While most symptom associations lacked statistical significance, consistent trends such as elevated tt-MA and MDA levels among symptomatic workers suggest possible relationships that may be masked by individual variability and sample size. For instance, workers with tremor exhibited the highest tt-MA levels, and those with fatigue showed nearly double the exposure of asymptomatic peers, warranting further investigation. The cross-sectional design also limits causal inference; longitudinal studies with larger and more diverse populations are needed to clarify exposure-symptom dynamics and capture spatial variability. Additionally, high smoking prevalence (82.5 %) may confound biomarker and symptom data, requiring adjusted models in future research.

### 3.11 Public health significance

The identification of specific biomarker-symptom associations in this informal sector population provides critical evidence for the health risks facing millions of workers in similar industries worldwide. The fact that measurable biological effects are occurring at current exposure levels, combined with the widespread exceedance of cancer risk thresholds demonstrated in our risk assessment, underscores the urgent need for intervention in this under-regulated sector.

Simple symptom screening, particularly for headache and appetite loss, could serve as a practical tool for identifying high-risk workers in resource-limited settings where sophisticated biomonitoring may not be feasible. The association between these symptoms and objective biomarker evidence provides confidence that such screening approaches could effectively identify workers requiring immediate attention or workplace modifications [59].

## 4 Conclusion

This study highlights significant occupational benzene exposure among informal footwear workers in Medan, evidenced by elevated ambient concentrations, biological markers, and associated health symptoms. The findings reveal that poor ventilation, long working hours, and high smoking prevalence contribute to a hazardous work environment, with several workshops exceeding international exposure and cancer risk thresholds. Biomarker analyses demonstrated consistent associations between benzene exposure, oxidative stress, and clinical symptoms such as headache and appetite loss, reinforcing the biological plausibility of adverse health effects.

Notably, oxidative stress markers (MDA) emerged as sensitive indicators of early neurological and metabolic disturbances, even when exposure levels were moderate. Given the unregulated nature of informal industries, these results underscore the urgent need for targeted interventions. Low-cost strategies such as improving natural ventilation, substituting hazardous adhesives with safer alternatives, promoting the use of personal protective equipment (PPE), and implementing biomarker-based screening could substantially reduce exposure and associated health risks in informal industrial settings. Future longitudinal studies and intervention trials are essential to guide policy and protect vulnerable worker populations in similar settings.

## Ethical Approval

This study was conducted in accordance with the Declaration of Helsinki and was approved by the Health Research Ethics Committee of the Faculty of Nursing, Universitas Sumatera Utara (Reference No. 1258/VI/SP/2017). Written informed consent was obtained from all participants prior to enrollment.

## Funding

The authors gratefully acknowledge financial support from the Research Institute of 10.13039/501100013375Universitas Sumatera Utara through the TALENTA Research Grant (Contract No. 5338/UN5.1.R/RPM/2017).

## Funding

This work was supported by the Research Institute of Universitas Sumatera Utara through the TALENTA Research Grant [Contract No. 5338/UN5.1.R/RPM/2017].

## CRediT authorship contribution statement

**Devi Nuraini Santi:** Writing – review & editing, Validation, Supervision, Resources, Project administration. **Evi Naria:** Investigation, Formal analysis, Data curation. **Taufik Ashar:** Writing – review & editing, Writing – original draft, Validation, Supervision, Software, Methodology, Conceptualization.

## Declaration of Generative AI and AI-assisted technologies in the writing process

During the preparation of this work the author used Microsoft Copilot in order to refine language, improve clarity, and structure responses to reviewer comments. After using this tool/service, the author reviewed and edited the content as needed and takes full responsibility for the content of the published article.

## Declaration of Competing Interest

The authors declare the following financial support: This work was supported by the Research Institute of Universitas Sumatera Utara through the TALENTA Research Grant (Contract No. 5338/UN5.1.R/RPM/2017), which covered research expenses and article publishing charges. The authors confirm that this financial support does not constitute a conflict of interest that could inappropriately influence, or be perceived to influence, the content or conclusions of this manuscript. No other competing financial interests or personal relationships exist among the authors.

## Data Availability

The data that has been used is confidential.
